# Supplementary material for: The effectiveness of a Malaysian House Officer (HO) preparatory course for medical graduates on self-perceived confidence and readiness: A quasi-experimental study
Source: PLoS One. 2020 Jul 17;15(7):e0235685. doi: 10.1371/journal.pone.0235685 (PMC7367441; doi:10.1371/journal.pone.0235685)
Supplement: S1 Table — (DOCX) [file pone.0235685.s001.docx]

**Table S1: Mean confidence levels for generic, practical task and personal skills at different time points**

| **Skills** |  | **Mean confidence level (SD) at baseline** |  | **Mean confidence level (SD) at post intervention** |  | **Mean confidence level (SD) at one month after working** |
| --- | --- | --- | --- | --- | --- | --- |
| **Generic Skills** | **Total, n** |  | **Total, n** |  | **Total, n** |  |
| Taking a history and performing relevant examination at first assessment of new admissions | 239 | 2.99 (2.19) | 224 | 3.46 (0.86) | 101 | 3.98 (0.71) |
| Make plan of management for new admissions | 239 | 2.10 (0.88) | 224 | 3.25 (0.79) | 101 | 3.52 (0.81) |
| Recognizing sick patients | 239 | 2.77 (0.93) | 223 | 3.64 (0.72) | 101 | 3.79 (0.73) |
| Functioning as a team member in assessing and managing sick patients | 238 | 2.74 (0.98) | 224 | 3.75 (0.73) | 101 | 3.81 (0.79) |
| Prioritizing and managing ward work | 239 | 2.33 (1.00) | 224 | 3.74 (0.79) | 101 | 3.88 (0.78) |
| **Practical tasks** |  |  |  |  |  |  |
| Starting resuscitation in hospital | 238 | 2.19 (0.82) | 224 | 3.61 (0.79) | 101 | 2.88 (0.99) |
| IV-line insertion (adult) | 239 | 2.57 (1.02) | 224 | 3.92 (0.86) | 101 | 4.17 (0.79) |
| Blood taking (adult) | 239 | 2.93 (1.09) | 224 | 4.15 (0.75) | 101 | 4.33 (0.73) |
| Inserting urinary catheter (male) | 239 | 2.47 (1.15) | 224 | 4.22 (0.72) | 101 | 4.07 (0.95) |
| Inserting urinary catheter (female) | 237 | 2.43 (1.12) | 224 | 4.36 (2.05) | 101 | 4.28 (0.81) |
| Do basic suturing and tie | 238 | 2.39 (1.01) | 222 | 3.91 (0.82) | 101 | 3.38 (1.01) |
| Prescribing common medications (format, not dosage) | 239 | 2.17 (0.93) | 223 | 3.21 (0.86) | 101 | 4.12 (0.80) |
| Requesting radiological investigations like CXR, CT | 238 | 2.08 (0.90) | 223 | 3.51 (0.84) | 101 | 3.95 (0.96) |
| Do a comprehensive review on patients during rounds | 238 | 2.05 (0.83) | 224 | 3.60 (2.14) | 101 | 3.94 (0.83) |
| Referring cases to another department | 239 | 1.83 (0.83) | 224 | 3.48 (0.84) | 101 | 3.72 (0.91) |
| Assisting operations | 239 | 1.94 (0.90) | 224 | 3.24 (0.84) | 101 | 3.63 (0.98) |
| Prescribing IV fluid (format of writing) | 239 | 1.81 (0.84) | 224 | 3.16 (0.89) | 101 | 3.64 (0.85) |
| Lumbar Puncture | 239 | 1.62 (0.76) | 133 | 2.26 (1.01) | 101 | 1.85 (0.92) |
| **Personal skills** |  |  |  |  |  |  |
| Team-working: e.g. sharing ward work, arranging rosters | 239 | 3.25 (1.09) | 224 | 3.93 (0.81) | 101 | 4.05 (0.85) |
| Handling criticisms from your senior colleagues | 238 | 3.06 (1.03) | 224 | 3.80 (0.83) | 101 | 4.03 (0.92) |
| Coping with additional, unexpected tasks | 239 | 3.08 (0.99) | 224 | 3.75 (0.80) | 101 | 3.90 (0.97) |
| Working independently away from home | 239 | 3.37 (1.05) | 224 | 3.68 (0.94) | 101 | 3.97 (1.01) |
| Referring cases to seniors | 238 | 2.76 (1.00) | 224 | 3.65 (0.79) | 101 | 4.07 (0.83) |

SD = Standard deviation
