# Supplementary material for: The effectiveness of a Malaysian House Officer (HO) preparatory course for medical graduates on self-perceived confidence and readiness: A quasi-experimental study
Source: PLoS One. 2020 Jul 17;15(7):e0235685. doi: 10.1371/journal.pone.0235685 (PMC7367441; doi:10.1371/journal.pone.0235685)
Supplement: S2 Table — (DOCX) [file pone.0235685.s002.docx]

**Table S2. Overall mean confidence and readiness scores at different time points**

|  | **Total, n** | **At baseline** | **Total, n** | **At post intervention** | **Total, n** | **At one month after working** |
| --- | --- | --- | --- | --- | --- | --- |
| Mean (SD) overall confidence scores | 238 | 2.16 (0.94) | 221 | 3.43 (0.75) | 101 | 3.78 (0.93) |
| Mean (SD) readiness scores | 238 | 2.26 (1.03) | 224 | 3.38 (0.83) | 101 | 3.71 (0.89) |

SD = Standard deviation
