# Supplementary material for: The effectiveness of a Malaysian House Officer (HO) preparatory course for medical graduates on self-perceived confidence and readiness: A quasi-experimental study
Source: PLoS One. 2020 Jul 17;15(7):e0235685. doi: 10.1371/journal.pone.0235685 (PMC7367441; doi:10.1371/journal.pone.0235685)
Supplement: S1 File — (PDF) [file pone.0235685.s003.pdf]

Part 1: Questionnaire Pre Medicorp HO Preparatory Course

Section A: SOCIO- DEMOGRAPHY

Please fill in the form below. Tick (/) where appropriate

PART A : PARTICIPANT'S DEMOGRAPHIC & WORKING BACKGROUND		
A1 - Age: ________ years		
A2 - Gender :	☐ Female	☐ Male
A3- Current marital status:	☐ Married	
	☐ Separated / Divorce	
	☐ Widow	
	☐ Never married/single	
A4- Religion:	☐ Islam	
	☐ Christian	
	☐ Hindu	
	☐ Buddha	
	☐ Others (Please specifiy; ______________	
A5- Ethnicity:	☐ Malay	
	☐ Chinese	
	☐ Indian	
	☐ Others (Please specify:__________)	
A6- Graduated from which University? __________________________<br>A7 -Graduation Year? ________________		

SECTION B: Clinical Experience

I have performed the following procedures roughly --- times THROUGHOUT MY UNDERGRADUATE YEARS (Please circle).

IV-line insertion in adult	None	1-5	5-10	10-20	>20
IV-line insertion in children/neonates	None	1-5	5-10	10-20	>20
Blood taking in adult	None	1-5	5-10	10-20	>20
Blood taking in children/neonates	None	1-5	5-10	10-20	>20
Giving IV medication	None	1-5	5-10	10-20	>20
Giving IM medication	None	1-5	5-10	10-20	>20
Giving PR medication	None	1-5	5-10	10-20	>20
Assisting all operations	None	1-5	5-10	10-20	>20
LP: assisting	None	1-5	5-10	10-20	>20
LP: performing	None	1-5	5-10	10-20	>20
Taking part in adult resuscitation	None	1-5	5-10	10-20	>20
Taking part in Paediatric/neonatal resuscitation	None	1-5	5-10	10-20	>20
Performing ECG	None	1-5	5-10	10-20	>20
Conducting vaginal delivery	None	1-5	5-10	10-20	>20
Inserting urinary catheter (male or female)	None	1-5	5-10	10-20	>20
Suturing (any minor or major surgery)	None	1-5	5-10	10-20	>20

Any other procedures ____

SECTION C: Confidence

1. On the following items, my level of confidence is: (all questions apply in general unless specified):

1 (totally not confident, even in theory); **2** (know only the approach in theory, not confident al all in actual practice)**:3** (only confident in making certain decisions, need seniors to be readily available/on constant standby); **4**(reasonably confident, need seniors at least being contactable for consultation); **5** (very confident, can be relied on without supervision)

Generic Skills

• Taking a history and performing relevant examination as first assessment of new admissions	1	2	3	4	5
• Make plan of management for new admissions	1	2	3	4	5
• Recognizing sick patients	1	2	3	4	5
• Functioning as a team member in assessing and managing sick patients	1	2	3	4	5
• Prioritizing and managing ward work	1	2	3	4	5

Practical tasks

1 (totally not confident, even in theory); **2** (know in theory but not confident al all in practice); **3** (know in theory, can perform some parts in practice independently, need supervision readily available/ on standby); **4** know in theory, confident in practice, need sources of supervision at least being contactable), **5** (know in theory, competent in practice without any supervision).

○ Starting resuscitation in hospital	1	2	3	4	5
○ IV-line insertion (adult)	1	2	3	4	5
○ Blood taking (adult)	1	2	3	4	5
○ Inserting urinary catheter (male)	1	2	3	4	5
○ Inserting urinary catheter (female)	1	2	3	4	5
○ Do basic suturing and tie	1	2	3	4	5
○ Prescribing common medications (format, not dosage)	1	2	3	4	5
○ Requesting radiological investigations like CXR, CT	1	2	3	4	5
○ Do a comprehensive review on patients during rounds	1	2	3	4	5
○ Referring cases to another department	1	2	3	4	5
○ Assisting operations	1	2	3	4	5
○ Prescribing IV fluid (format of writing)	1	2	3	4	5
○ Lumbar Puncture	1	2	3	4	5

Soft Skills

1. Not confident at all, 2. Little confidence, 3. Neither, 4. Somewhat confident, 5- very confident

I feel confident about my ability in…

- Team-working: e.g. sharing ward work, arranging rosters
  Finding out the ward routines/protocols on your own 1 2 3 4 5
- Handling criticisms from your senior colleagues 1 2 3 4 5
- Coping with additional, unexpected tasks 1 2 3 4 5
  Managing own time on and off work
- Working independently away from home 1 2 3 4 5
- Referring cases to seniors 1 2 3 4 5

4. If I am to report for work as a house officer tomorrow, my level of confidence to work as a house officer is:

Not confident at all Very Confident

1----------------- 2-----------------3----------------- 4----------------------5

5. In my opinion, the most daunting aspect of being a house officer is: (Please mark ONLY ONE)

1. Physical demand: difficulty in maintaining patience
2. Physical demand: difficulty in maintaining clinical judgement
3. Confronting the seniors
4. Handling patients with responsibilities (including communication)

5. Competence required in knowledge and judgement
6. Competence required in practical skills
7. Time management
8. Having to adjust to different routines in work and life

Others: ____

SECTION D: Readiness

If I am to report for work as a house officer tomorrow, my level of readiness is:

(Please circle response on the scale below)

Far from ready Looking forward to it

1----------------- 2-----------------3----------------- 4----------------------5

THANK YOU FOR YOUR TIME
