# Supplementary material for: The effectiveness of a Malaysian House Officer (HO) preparatory course for medical graduates on self-perceived confidence and readiness: A quasi-experimental study
Source: PLoS One. 2020 Jul 17;15(7):e0235685. doi: 10.1371/journal.pone.0235685 (PMC7367441; doi:10.1371/journal.pone.0235685)
Supplement: Attachment — Submitted filename: response to reviewers comments HO prep .docx [file pone.0235685.s005.docx]

Aneesa Abdul Rashid

Family Medicine Department,

Faculty of Medicine and Health Sciences,

Universiti Putra Malaysia (UPM),

Serdang 43400 Selangor,

Malaysia

aneesa@upm.edu.my

Editor-in-Chief

PLOS-ONE

May 30, 2020

Dear Editor-in-Chief,

It is our pleasure to submit the revised version of our manuscript entitled “**The Effectiveness of a Malaysian House Officer (HO) Preparatory Course for Medical Graduates on Self-Perceived Confidence and Readiness: A Quasi-Experimental Study**” by Aneesa Abdul Rashid, Sazlina Shariff Ghazali, Iliana Mohamad, Maliza Mawardi, Husna Musa, and Dalila Roslan.

Below is a table addressing the editor and reviewers’ comments. We have done our best to update it according to the requirement of the reviewers and journal.

Thank you for the constructive comments for improvement

Sincerely,

Aneesa Abdul Rashid

Family Medicine Specialist & Medical Lecturer

Department of Family Medicine

Universiti Putra Malaysia (UPM)

| Editor, reviewers’ comments. | Authors’ comments | Page, Line, File |
| --- | --- | --- |
| **Editor & Journal Requirements** |  |  |
| To enhance the reproducibility of your results, we recommend that if applicable you deposit your laboratory protocols in protocols.io, where a protocol can be assigned its own identifier (DOI) such that it can be cited independently in the future. For instructions see: <http://journals.plos.org/plosone/s/submission-guidelines#loc-laboratory-protocols> | Thank you for your suggestion, however our protocol is already published at: <https://bmjopen.bmj.com/content/9/8/e024488>  It is registered in <https://bmjopen.bmj.com/content/9/8/e024488>  It is also attached as S2 File | File S2 |
| 1. Please ensure that your manuscript meets PLOS ONE's style requirements, including those for file naming. The PLOS ONE style templates can be found at https://journals.plos.org/plosone/s/file?id=wjVg/PLOSOne_formatting_sample_main_body.pdf and https://journals.plos.org/plosone/s/file?id=ba62/PLOSOne_formatting_sample_title_authors_affiliations.pdf | We have done our best to ensure that the amended manuscript meet the PLOS ONE’s style. |  |
| 2. PLOS ONE will consider submissions that present new methods, software, or databases as the primary focus of the manuscript if they meet the criteria of utility, validation, and availability described here: http://journals.plos.org/plosone/s/submission-guidelines#loc-methods-software-databases-and-tools. To meet these criteria, please provide supporting materials enabling other teachers and researchers to replicate your teaching intervention such as sample worksheets, a detailed lesson plan or curriculum or other educational materials. If you include supporting materials, they should not be under a copyright more restrictive than CC-BY. | Thank you for your suggestions. We attach our questionnaire (S1 file), and also the details of the module and timeline of the course is published in the protocol paper as mentioned above (S2 file) | File S1  File S2 |
| 3. We note that you have indicated that data from this study are available upon request. PLOS only allows data to be available upon request if there are legal or ethical restrictions on sharing data publicly. For information on unacceptable data access restrictions, please see http://journals.plos.org/plosone/s/data-availability#loc-unacceptable-data-access-restrictions. | Thank you for highlighting about the data availability as required by PLOS One. We have included 2 supporting tables to describe the data we used in this study (S1 & S2 Tables). However, we did not put these supporting tables in our manuscript. At present, we are not able to share the raw data as we are doing more ongoing data analysis for upcoming publications related to this research project. | Table S1  Table S2 |
| a) If there are ethical or legal restrictions on sharing a de-identified data set, please explain them in detail (e.g., data contain potentially identifying or sensitive patient information) and who has imposed them (e.g., an ethics committee). Please also provide contact information for a data access committee, ethics committee, or other institutional body to which data requests may be sent.  b) If there are no restrictions, please upload the minimal anonymized data set necessary to replicate your study findings as either Supporting Information files or to a stable, public repository and provide us with the relevant URLs, DOIs, or accession numbers. Please see http://www.bmj.com/content/340/bmj.c181.long for guidelines on how to de-identify and prepare clinical data for publication. For a list of acceptable repositories, please see http://journals.plos.org/plosone/s/data-availability#loc-recommended-repositories.  We will update your Data Availability statement on your behalf to reflect the information you provide. | Answered as above |  |
| 4. We note you have included a table to which you do not refer in the text of your manuscript. Please ensure that you refer to Table 3 in your text; if accepted, production will need this reference to link the reader to the Table. | We have made the relevant corrections, thank you for pointing out the error in type. |  |
| 5. Please include your tables as part of your main manuscript and remove the individual files. Please note that supplementary tables (should remain/ be uploaded) as separate "supporting information" files | We have done as per requested |  |
| **Reviewer 1** |  |  |
| This study explores the effectiveness of a HO preparation course conducted for newly qualified doctors in Malaysia, comparing confidence, readiness for practice and psychological well-being. | Thank you for your kind comments, we have tried to address all your comments as best we could |  |
| On the whole the findings seem to support the authors conclusions although they rightly include in the limitations of the study, the lack of a control group and the large loss to follow-up at 1 month post-employment. | It was most challenging to follow up the young doctors as mentioned in our limitations, this included the busy schedule and also the large amount of administrative work of following up each hospital to request permission on follow up of the participants. |  |
| The study includes a high proportion of the participants who qualified overseas (although it is not stated where) which it may be worth reflecting in their conclusions. | Thank you for this suggestion, we have added on this to conclusion and in the abstract | Page 3, Line 53 |
| - The authors state they can not publish the data due to personal information it contains. Is it not possible to publish an anonymised version of the data set, as happens with many other studies? | Thank you for highlighting about the data availability as required by PLOS One. We have included 2 supporting tables to describe the data we used in this study (S1 & S2 Tables). However, we did not put these supporting tables in our manuscript. At present, we are not able to share the raw data as we are doing more ongoing data analysis for upcoming publications related to this research project. | Page 20, Line 411 |
| - The use of English could be improved generally but particularly in lines 103-104 and 280-281. | We have corrected the grammar on the mentioned lines and also the article as a whole | Page 4, Line 81  Page 14, Line 283 |
| - The table attribution in line 241 is incorrect. | We have corrected it and attributed table 3 and 4 in their rightful position | Page 12, Line 239  Page 13, Line 245  Page 13, Line 248  Page 13, Line 254 |
| - In line 272, the authors describe a minimum confidence level require for final year students. It would be beneficial to clarify this point including who sets the minimum, are the students/newly qualified doctors aware of this? | The original authors of the tool used to assess confidence and readiness among senior students, had decided the level of confidence to be a minimum level of ‘3’ for their final year students. This was the standard set in a local Malaysian University [15]. We did not disclose this to the participants as we did not want this information to effect their response to the questionnaire. | Page 7, Line 161 |
| - There is not mention of the cost of the course, just that it is now a private enterprise.  - Can they comment on the cost of doing the course and any impact this might have on the recruitment or findings of the current study. | Thank you for this comment, we agree on this statement and have included explanations as below:  **Materials & Methods/ outcome measures:**  The course charges RM 450 (USD 100) for a three-day course this includes cost of venue, refreshments and food, training facilities such as medical equipment and mannequins, and trainer fees. The cost of running this HO preparatory course is around RM 350-400 per person  **Strengths and limitations:**  It is also important to mention that this is a paid course, thus limiting the participants to those that have the financial means | Page 6, Line 135  Page 16, Line 316 |
| **Reviewer 2** |  |  |
| This is an interesting and well written paper that makes a valuable contribution. | Thank you for the encouraging comment, we feel that interventions such as this which is highly popular here, needs an evidence-based assessment. |  |
| I would be interested to know if House Officers were provided with a formal induction by the hospitals they worked in and if so what the nature of the induction was as this may have had a bearing on the scores one month after commencing work. | From the feedback of the alumni, all hospitals would have their formal induction for house officers but is mainly on administrative issues and not on the hands-on experience itself.  We do acknowledge this as one of the potential bias of the follow up cohort and can be considered in future experiments which we recommend looking into control groups (mentioned in conclusion and recommendation)  We have also added this point in ‘**strength and limitations’**:  Lastly, there are many factors that may affect the confidence, readiness and psychological well-being post working one-month including hospital HO inductions and the social support of the participants | Page 16, line 318 |
| The paper would also be improved by providing a more detailed description as to how the skills were taught, for example, were models used to teach practical skills. The topics covered seem a lot for a three day course and it would be interesting to understand a bit more as to the timetable and structure of the course. | Thank you for the comment, the details of this course is published in a paper entitled Quasi-experimental study on the effectiveness of a house officer preparatory course for medical graduates on self-perceived confidence and readiness: a study protocol  We have attached this as file as additional file S1 for your reference | S1 File |
| I am concerned about making direct comparisons with the 1 month after starting score as the numbers returning the survey were less than half. | We encountered many challenges in the follow up of this experiment. We did our best and invested tremendous efforts to follow up the participants in which we mentioned in **‘material and methods’**  The participants were initially reminded of their participation on this study via WhatsApp messages, which was part of the effort to facilitate the module a few days before making the call. The participants were then followed up via calls after office hours. If there was no response, another two reminders via personal messages was sent approximately one week apart. Participants would reply the messages on which the appropriate dates were available to be interviewed. | Page 5, Line 103 |
| But nevertheless this paper makes a meaningful contribution to an important subject | Thank you for your encouraging remark, we hope that this will help facilitate future efforts to improve medical training in the near future. |  |
